# The Effects of Clinical Applications of Robot-Assisted Therapy Methods: End-Effector, Fixed Exoskeleton, and Wearable Exoskeleton on Functional Activities in Stroke Patients

**DOI:** 10.3390/life16030396

**Published:** 2026-02-28

**Authors:** Jung-Ho Lee

**Affiliations:** Department of Physical Therapy, University of Kyungdong, 815 Gyeonhwon-ro, Munmak-eup, Wonju-si 26495, Gangwon-do, Republic of Korea; ljhcivapt@naver.com

**Keywords:** robot, stroke, functional activity, gait, balance

## Abstract

*Background and Objectives*: This study was conducted to investigate the effects of robot-assisted gait rehabilitation approaches using commonly used end-effector, fixed exoskeleton, and wearable exoskeleton on gait and balance abilities in patients with early post-stroke (≤3 months). *Materials and Methods*: Sixty patients admitted to a rehabilitation center with confirmed stroke by a medicine specialist were assigned to three groups such as the end-effector group (EG 1), the fixed exoskeleton group (EG 2), and the wearable exoskeleton group (EG 3). The primary endpoint was pre-specified as the change in timed up-and-go gait test (TUG) from baseline to week 6, and all other outcomes were treated as secondary. The functional gait category (FAC), 10-m walk test (10MWT), six-minute walk test (6MWT), timed up-and-go gait test (TUG), dynamic gait index (DGI), and Berg Balance Scale (BBS) were measured at four time points (baseline, 2 weeks, 4 weeks, and 6 weeks). *Results*: A significant main effect of time was observed for all outcome variables, but neither the main effect of group nor the interaction between group and time was significant for any outcome variable. Within-group analyses revealed that FAC, 6MWT, DGI, and BBS increased over time in all groups, whereas 10MWT and TUG decreased. *Conclusions*: All three robot-assisted gait rehabilitation approaches in patients with early post-stroke were associated with significant improvements in gait and balance abilities over 6 weeks. However, statistically significant differential trajectories were not detected across robot types in this sample.

## 1. Introduction

Stroke remains a leading cause of mortality and long-term disability worldwide, and its prevalence and socioeconomic burden continue to rise with population aging. Many stroke survivors experience persistent impairments such as hemiparesis, muscle weakness, impaired coordination, sensory deficits, and cognitive dysfunction. These sequelae substantially restrict the performance of fundamental functional activities, including walking, transfers, and upper-limb use for activities of daily living (ADL) [1]. Such limitations extend beyond reduced physical capacity and are closely associated with diminished quality of life, delayed community and vocational reintegration, and increased reliance on long-term care [2]. Accordingly, restoration of functional activities is regarded as a primary determinant of prognosis in stroke rehabilitation, reinforcing the need to develop effective and scalable rehabilitation strategies [3].

Conventional rehabilitation in clinical settings typically includes individualized therapeutic exercise, gait training, balance training, task-oriented training, and ADL practice delivered by physical therapists. These interventions have demonstrated meaningful benefits for functional recovery and remain a cornerstone of stroke rehabilitation [4]. However, traditional approaches are inherently constrained by therapist-dependent variability and limited human and time resources, which restrict the achievable training intensity and repetition volume [5]. Although high-intensity, high-frequency repetitive practice is recommended from a neuroplasticity perspective, maintaining this intensity and frequency in everyday clinical settings is difficult. Fatigue in both patients and therapists frequently limits prolonged, intensive training [6]. In addition, for patients with severe motor deficits or poor balance, fall prevention and safety management may require two or more therapists, increasing staffing demands. Practical constraints such as treatment space, equipment availability, and patient throughput further reduce the treatment time that can be allocated per patient, limiting the total amount of training required to maximize functional recovery [7].

Another limitation of conventional therapy is the difficulty of objectively quantifying and standardizing intervention dose and performance. Therapists often adjust task difficulty and intensity based on clinical judgment, but systematic recording of repetitions, movement quality, and real-time feedback is frequently insufficient [8]. This restricts the precision of individualized prescriptions aligned with each patient’s recovery trajectory and functional level, and it also complicates the quantification and comparability of intervention intensity in research contexts. Therefore, there is an increasing demand for rehabilitation platforms capable of delivering high-dose training while enabling objective monitoring, standardized dosing, and data-driven feedback. In this context, robot-assisted therapy has been proposed as a clinically applicable approach to provide repetitive, intensive, and measurable training [9,10].

Against this backdrop, robot-assisted therapy has emerged as a promising treatment paradigm for improving functional recovery after stroke. Robotic rehabilitation is designed to provide high-volume, task-specific, repetitive training by guiding the patient’s limb movements through programmable mechanical assistance [11]. Compared to conventional therapy, robotic systems can provide longer training times and more repetitions while maintaining safety and consistency [12]. Specifically, robotic devices can generate consistent and reproducible movement patterns based on preset parameters such as trajectory, joint torque, and assistance or resistance levels, facilitating the standardization of treatment. This standardization allows for systematic control of training volume at the individual patient level and objective monitoring of actual performance according to predefined training plans [13].

From a neuroplasticity perspective, repetitive and intensive training is considered a key driver of neural reorganization and functional recovery, promoting adaptive reorganization of damaged neural circuits and strengthening associated sensorimotor pathways [14]. Based on these principles, robot-assisted rehabilitation aims to maximize motor learning by safely increasing the number of repetitions in a controlled environment [15]. Furthermore, robotic training can promote error-based learning by allowing patients to repeatedly identify performance errors and adjust their movement strategies through practice and feedback. This characteristic can be particularly beneficial for relearning functional movement patterns that underlie real-life activities, beyond improving individual functional impairments such as muscle strength or joint range of motion [16].

However, robotic-assisted therapy is not a single, uniform approach. Robotic devices vary significantly in mechanical design and clinical application [17]. Specifically, three types are widely used in clinical settings: end-effector systems [18], fixed exoskeletons [19], and wearable exoskeletons [9]. Each type may have different task constraints, levels of support, and ecological validity, which may lead to differences in their impact on the functional activities of stroke survivors. However, most studies have compared single or two robots or conducted only fragmentary evaluations. Furthermore, few studies have examined changes in functional activities associated with each robot through continuous assessment [20]. Therefore, this paper aims to compare the clinical effects of three robot-assisted treatment methods (end-effector, fixed exoskeleton, and wearable exoskeleton) on the functional activities of stroke patients and to provide a basis for device selection and application in clinical rehabilitation.

## 2. Materials and Methods

### 2.1. Subjects

This study involved patients admitted to the rehabilitation medicine department of a single institution. A total of 60 patients diagnosed with ischemic stroke by magnetic resonance imaging were included. The diagnosis was made by a rehabilitation medicine specialist. Participants were assigned to three groups based on the type of robotic therapy device, namely end-effector group, fixed exoskeleton group, and wearable exoskeleton group, based on a computer-generated sequence.

All participants received a detailed explanation of the study’s purpose, procedures, subjects’ rights, potential side effects, and compensation before participation. This study was conducted in accordance with the Declaration of Helsinki. All procedures and protocols were reviewed and approved by the Institutional Review Board, and the study was conducted in compliance with the committee’s guidelines (1041107-202404-HR-010-02, Approval date: 17 May 2024). This study was not prospectively registered in a clinical trial registry. The absence of trial registration is acknowledged as a limitation.

Patients were included if they had a confirmed stroke on neuroimaging within 3 months of onset, could understand and follow simple instructions, could stand with assistance or partially bear weight during supervised ambulation, and met the size and weight requirements of the device. Patients were excluded if they had a history of other neurological disorders that could affect motor recovery, severe musculoskeletal conditions that could limit lower extremity training, or conditions that increased exercise risk, such as unstable cardiovascular disease, severe respiratory disease, or severe orthostatic hypotension. Additionally, patients were excluded if they had severe cognitive impairment or aphasia that could impede safe participation, severe spasticity or contractures that could limit device use, or severe skin conditions at the contact site.

### 2.2. Design

Before baseline assessment, the 60 participants who met the inclusion/exclusion criteria were randomized into three groups, the participants who met the inclusion/exclusion criteria were randomized into three groups: the end-effector group (EG1), the fixed exoskeleton group (EG2), and the wearable exoskeleton group (EG3). Participants were assigned in a 1:1:1 ratio using a computer-generated random sequence (simple randomization) created prior to enrollment. No stratification was used. Allocation was implemented after completion of baseline assessments to minimize selection bias.

In this study, all three groups of subjects received traditional rehabilitation treatment. Traditional rehabilitation treatment consisted of physical therapy and occupational therapy prescribed by a rehabilitation medicine specialist. Physical therapy included gait and balance training, lower extremity muscle strength and endurance training, and trunk stability training. Occupational therapy included upper extremity functional training, activities of daily living training, and cognitive and perceptual function training, tailored to the patient’s condition. Traditional rehabilitation treatment was performed twice a day, 30 min per session, 5 days a week, for a total of 6 weeks. Robot-assisted therapy was applied after traditional rehabilitation treatment, depending on the type of robot used. Before the initial robot application, participants were educated on the overall application method, principles, and purpose of the robot. Afterwards, rehabilitation training was conducted using the robot assigned to each group, once daily for 40 min, 3 days a week for a total of 6 weeks. During robot-assisted gait training, participants were monitored for tolerability and potential adverse events, including fatigue, soreness/pain, dizziness, and skin condition. Sessions were paused if clinically indicated based on participant symptoms or therapist judgment. The study followed a 6-week intervention timeline. Outcome assessments were performed at baseline and repeated at weeks 2, 4, and 6. Throughout the intervention time, participants in all groups received conventional PT/OT, and additionally participated in robot-assisted gait training according to assigned device type. The assessment schedule and intervention time were identical across groups.

**Figure 1:**
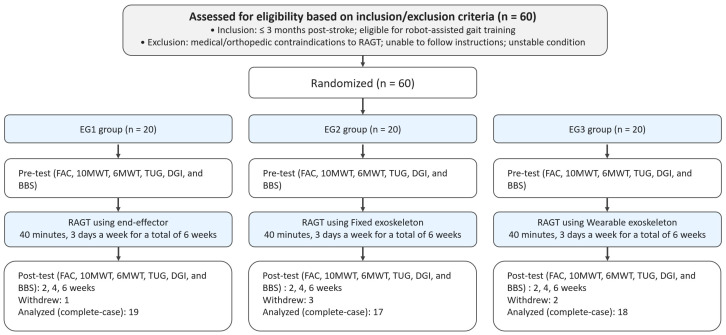
Participant flow diagram showing enrollment, allocation, follow-up at each assessment time point, and analysis, including reasons for withdrawal.

In this study, to compare the effects of the robots applied to each group, functional activity assessments such as the functional ambulation category (FAC), 10-m walk test (10MWT), six-minute walk test (6MWT), timed up-and-go test (TUG), dynamic gait index (DGI), and Berg Balance Scale (BBS) were used. Evaluations were conducted before the initial application of the robot intervention, and after 2, 4, and 6 weeks, and all evaluations were conducted by independent evaluators not directly involved in the robot intervention (Figure 1).

### 2.3. Intervention

To ensure comparability between devices in dose standardization and clinical titration, the scheduled time of robot-assisted training was standardized to 40 min per session across all groups (same frequency and total intervention duration). In routine clinical practice, however, device parameters (speed, body-weight support, and assistance level) were individualized by therapists based on safety and patient capability. Treating therapists adjusted key device parameters based on each patient’s motor capacity, balance, fatigue, and safety. Because parameter settings were titrated dynamically and device-exported dose logs were not consistently available across all platforms and sessions, complete objective dose metrics could not be uniformly extracted for all participants.

The EG1 group was equipped with an end-effector robot device (MW-S200, Curexo, Seoul, Republic of Korea). The end-effector gait training session lasted 40 min, consisting of a five-minute preparation phase (harness application, standing, and safety check), a 30-min main training phase (actual robotic walking), and a five-minute cool-down phase (speed reduction, harness release, and sitting rest). In the initial session, walking speed was set within the range of 0.3~0.5 m/s, considering the subjects’ baseline walking ability and cardiovascular status, and the weight-bearing ratio was initially set at 30~40%.

The therapist assessed the subjects’ subjective fatigue, dizziness, or chest pain during each session and adjusted the training intensity by gradually increasing the speed by 0.1~0.2 m/s and decreasing the weight-bearing ratio in 5% increments within a safe range. During end-effector training, verbal feedback was provided regarding toe-off, heel-strike, and weight-shift symmetry within the gait cycle. When necessary, the subjects were instructed to hold a handle with the unaffected upper limb to assist trunk stability. If the subject complained of severe fatigue or observed cardiovascular signs, the speed was immediately reduced or stopped, and if necessary, the robot training for that day was stopped.

Subjects in the EG2 group underwent training using a fixed exoskeleton robot device (Lokomat Nanos, Hocoma AG, Zurich, Switzerland). Participants in the fixed exoskeleton group underwent gait training using a lower exoskeleton robot fixed to a treadmill and a weight-bearing device. The subjects stood in front of the device and donned the exoskeleton, ensuring that the robot joints corresponding to the hip, knee, and ankle joints were aligned with the human joint axes as closely as possible. Fixation bands were used to secure the exoskeleton to the thigh and lower leg. The exoskeleton was then connected to the weight-bearing device via an upper harness to ensure standing stability. During initial setup, the therapist assessed the range of motion of the lower extremity joints, the degree of stiffness, and the presence of joint pain. The therapist then individually adjusted the allowable range of motion for each joint and the assistive torque level of the robot.

Each session of the fixed exoskeleton consisted of five minutes of standing and acclimation (wearing the harness and exoskeleton, checking joint alignment, and demonstrating low-speed walking), 30 min of main training (repeating a normative gait pattern or active gait training), and five minutes of cool-down (reducing speed and assistance, transitioning from standing to sitting). The initial walking speed was set at 0.3~0.5 m/s. Prioritizing joint alignment and gait pattern stability, the focus was on reducing assistance torque and inducing active muscle contraction over increasing speed for a certain period. The weight-bearing ratio was typically started at 30~50% and decreased in 5~10% increments as the subjects gradually adapted to standing and walking, with care taken to avoid excessive load on the paretic lower extremity.

In the fixed exoskeleton group, the initial session focused on normalizing joint alignment and gait pattern. The therapist provided verbal feedback on hip extension, knee flexion/extension timing, trunk forward tilt, and pelvic alignment during the gait cycle, allowing for upper extremity support (parallel bars, handrails) when necessary. Subjective fatigue and muscle soreness were monitored throughout all sessions, and skin damage around the robot-worn area was checked before and after each session. If any abnormalities were found, immediate rest was recommended.

The EG3 group received intervention using a wearable exoskeleton robot device (ANGEL LEGS M20, Angel Robotics, Seoul, Republic of Korea). Subjects in the wearable exoskeleton group underwent overground gait training using a wearable lower exoskeleton with integrated power and control units. The subjects wore exoskeleton frames on their waists, thighs, and lower legs, and the drive modules at the hip and knee joints were adjusted to their body shape. After fitting the device, they typically used a walking assistive device, such as a walker or quadruped walker, to ensure initial gait stability. The robot’s assistance mode was set to start in full-assist mode and gradually transition to partial-assist mode as the subjects’ active participation improved.

The wearable exoskeleton training sessions were divided into a preparation phase, core gait training phase, functional task phase, and cool-down phase, each lasting a total of 40 min. The preparatory phase involved donning the equipment, safety checks, and simple joint mobilization and weight-bearing exercises (five minutes) in a sitting or standing position. The core gait training phase (20 min) focused on straight-line walking and turning gait on a flat surface in the ward corridor or rehabilitation room. Initially, the training consisted of intermittent walk–rest intervals, with repeated 10~15-m segments. The goal was to gradually increase the total walking distance per session to 100~300 m, as long as the subjects’ cardiorespiratory capacity and gait stability permitted. The functional task phase (10 min) included real-life tasks such as turning, obstacle avoidance, speed changes, and short stair climbs to promote functional gait adaptation. A cool-down phase (speed reduction, harness release) lasted approximately five minutes.

Each session was individualized by adjusting the level of robotic assistance, walking speed, walking distance, and assistive device dependence. For example, if there was no risk of falling for a certain period and gait was maintained stably, the difficulty level was gradually increased by changing the assistive device from a walker to a cane or reducing the therapist’s physical protection. Since fall prevention was particularly important in the wearable exoskeleton group, all sessions were conducted by a physical therapist with sufficient robot use experience, with the subjects’ sides and backs protected. Uneven floors and crowded hallways were avoided. Abnormal findings such as lower extremity pain, joint discomfort, dizziness, and palpitations were checked before and after the session, and if any abnormalities were found, the intensity of the session was adjusted or the training was stopped.

Session duration and schedule were standardized across groups; however, key intensity parameters (walking speed, body-weight support, and assistance level) were individualized by therapists according to patient safety and capability. Device-exported dose logs were not consistently retrievable from routine clinical practice; therefore, detailed objective dose metrics could not be reported in a reliable and complete manner.

### 2.4. Assessment Method

In this study, functional walking level, walking speed, walking endurance, walking stability, and balance ability were evaluated to understand the functional recovery of early post-stroke (≤3 months) patients according to robot-assisted treatment methods.

The functional ambulation scale was used to assess functional walking ability. The FAC is a clinical assessment tool that categorizes functional walking ability into six levels (0~5) based on the degree of assistance required for walking. The assessment was performed by a therapist observing the subject’s gait and focused on the need for human assistance, regardless of the use of walking aids. Scores range from 0 (unable to walk or requiring assistance from two or more people) to 5 (able to walk independently on all surfaces), with a score of 4 indicating independent walking on level ground and a score of 5 indicating independent walking in all environments [21].

In this study, the 10MWT was used to measure walking speed. The 10MWT is a test that measures short-distance walking speed. To ensure standardization, a straight path was secured, and only the central 10-m section of a 14-m path (including two-meter at each end) with acceleration and deceleration sections was used as the time measurement section. Subjects were instructed to walk at their usual walking pace. Participants were allowed to use their usual walking aids, and the same assistive device was used consistently across all assessment time points. In addition to 10MWT, walking speed (m/s) was calculated as speed = 10 m/time (s) for clinical interpretation and sensitivity analysis [22]. The 6MWT used to assess walking is an assessment that assesses walking endurance/functional exercise capacity by asking subjects to walk as far as possible in six minutes. The total walking distance (meter) was used as the measurement value for the study results. A 30-m straight section was set, with regular intervals and clearly defined turning points before the assessment. Subjects were instructed to walk as far as possible in six minutes, prioritizing safety and resting when necessary. Assistive devices were permitted, but the test was conducted under identical conditions [23]. The TUG test used in this study assesses basic functional mobility. It measures the time it takes to rise from a chair, walk three meters, and then return to a sitting position. Participants began the test seated on a standard-height armchair and were instructed to perform the test at a comfortable and safe pace. The test began when the participant’s buttocks lifted off the chair according to the evaluator’s verbal instructions and ended when the participant’s buttocks re-attached to the chair. The test was recorded in seconds [24].

The DGI used in this study is a tool that assesses dynamic balance and gait adaptability to various environmental demands during walking. This study utilized eight items on the DGI, each scored on a four-point scale ranging from 0 to 3, resulting in a total score of 0 to 24. Higher scores indicate greater gait stability and adaptability. The assessment was conducted on a level, safe walking path. Subjects performed each item according to the examiner’s instructions, and the examiner observed the subjects’ performance and assigned scores based on the criteria for each item [25]. Balance ability was assessed using the BBS. The BBS is a 14-item scale assessing standing and postural control. Each item is rated on a five-point scale ranging from 0 (unable to perform) to 4 (normal performance), with a total score ranging from 0 to 56. Items include sit-to-stand, static standing, standing with eyes closed, standing with feet together, reaching forward, picking up an object, turning, tandem stance, and standing on one leg. Each item was performed according to standardized instructions, and scores were assigned based on observation of the subject’s performance time, the need for assistance, and the degree of postural sway. Furthermore, for safety reasons, the evaluator positioned himself either to the side or behind the subject during the assessment to prevent falls [26]. Outcome assessments were performed by an assessor who was blinded to group allocation. To maintain blinding at all time points, allocation information was not disclosed to the assessor, and participants were instructed not to discuss their intervention during assessments.

### 2.5. Statistical Analysis

The statistical program SPSS PC for Windows (version 18.0) was used for all variable data obtained through this study, and the mean and standard deviation were calculated through descriptive statistics. The Shapiro–Wilk test was used to test for normality of the evaluation items, and one-way ANOVA was used to examine the general characteristics of the subjects and the homogeneity between groups. The primary endpoint was pre-specified as the change in TUG from baseline to week 6, and all other outcomes were treated as secondary. Fisher’s LSD post hoc comparisons were conducted only following significant omnibus effects and were interpreted as exploratory. To examine changes in each evaluation item over time and differences between groups, a repeated-measures ANOVA was performed, with group as the between-group factor and time as the within-group factor. The primary repeated-measures analyses were performed as complete-case analyses because repeated-measures ANOVA requires complete data across all assessment time points. No imputation was applied. Effect sizes are reported as partial eta squared (ηp^2^) for omnibus tests. Values for the group and group-by-time terms may be small when trajectories are similar across groups despite large time effects. The sphericity assumption was evaluated using Mauchly’s test. When sphericity was violated, degrees of freedom were adjusted using the Greenhouse–Geisser correction. Because repeated-measures ANOVA requires complete observations across assessment time points, the primary analysis was conducted as a complete-case analysis including only participants with complete repeated measurements, and no imputation was applied. When omnibus tests were significant, Fisher’s LSD was used for post hoc comparisons (α = 0.05). An a priori sample size estimation was conducted using G*Power (version 3.1.9.7; Heinrich Heine University Düsseldorf, Düsseldorf, Germany) for a mixed repeated-measures design with three groups and four measurement time points (pre-test, week 2, week 4, and week 6). Based on an assumed small-to-moderate effect size (*f* = 0.19), a two-sided alpha of 0.05, and power of 0.80, the minimum required total sample size was 50 participants. To account for expected attrition in early post-stroke rehabilitation, 60 participants were enrolled and randomized (1:1:1). The primary endpoint was defined a priori as the change in TUG from baseline to week 6.

## 3. Results

All groups initially enrolled 20 participants. During follow-up, one participant in EG1, three in EG2, and two in EG3 discontinued participation due to personal reasons. Therefore, the repeated-measures analyses included 19 participants in EG1, 17 in EG2, and 18 in EG3. Data from participants who discontinued were not used in the analyses at any time point, including the pre-test. Thus, all statistical analyses were conducted using only participants with complete repeated measurements. In this study, age, height, and weight were used to determine the general characteristics of the subjects. The initial assessment of the evaluation items and general characteristics were used to test for homogeneity. Table 1 below shows the general characteristics and pre-test homogeneity of the three groups. There were no significant differences between groups in age, height, weight, MMSE, onset time, stroke type, lesion side, NIHSS, FMA-LE, MAS, FAC, 10MWT, 6MWT, TUG, DGI, and BBS. Because device-exported dose logs were not consistently available, objective dose metrics could not be summarized quantitatively.

In all repeated-measures analyses, time refers to the four assessment points (pre-test, weeks 2, 4, and 6). Repeated-measures ANOVA on TUG showed a significant main effect of time (*F* = 18.193, *p* < 0.001) (Table 2). On the other hand, the main effect of group was not significant (*F* = 0.023, *p* = 0.977), and the group × time interaction was also not significant (*F* = 0.222, *p* = 0.969). Consistent with the non-significant group and interaction terms, ηp^2^ values for group and group × time effects were small, reflecting similar change trajectories across robot types. No statistically significant differences were observed in the TUG comparison between groups at each evaluation time.

Within-group changes were examined, and repeated-measures ANOVAs were significant in EG 1 and EG 3 (EG 1: *F* = 36.050, *p* < 0.001; EG 3: *F* = 14.128, *p* < 0.001). In contrast, EG 2 did not reach significance (*F* = 2.514, *p* = 0.069) (Table 3). Post hoc tests showed that in EG 1, pre-test scores were significantly higher than weeks 2, 4, and 6. Week 2 scores were significantly higher than weeks 4 and 6. Week 4 scores were significantly higher than week 6, confirming a gradual decline over time. In EG 2, pre-test scores were significantly higher than weeks 2, 4, and 6. Scores from weeks 2 and 4 were significantly higher than week 6. In EG 3, the dictionary was significantly higher than weeks 2, 4, and 6, and week 2 was significantly higher than weeks 4 and 6.

A repeated-measures ANOVA on the FAC, conducted to assess the subjects’ functional walking ability, revealed a significant main effect of time (*F* = 80.528, *p* < 0.001). However, the main effect of group was not significant (*F* = 0.382, *p* = 0.684), and the group × time interaction was also not significant (*F* = 0.236, *p* = 0.921) (Table 4). Additionally, no statistically significant differences were observed in the FAC comparison between groups at each evaluation time.

Analysis of within-group changes revealed significant differences across all groups in a repeated-measures ANOVA (Table 5). The *F*-value for EG 1 was 27.530 (*p* < 0.001), 24.920 (*p* < 0.001) for EG 2, and 28.680 (*p* < 0.001) for EG 3, indicating that FAC scores improved over time in all three groups. Post hoc analyses revealed similar patterns across all three groups. That is, the FAC score significantly increased at 2, 4, and 6 weeks compared to the pre-test, and a significant increase was confirmed at 4 and 6 weeks compared to 2 weeks; a significant increase was also confirmed at 6 weeks compared to 4 weeks, indicating that the FAC tended to improve gradually as the evaluation time progressed.

A repeated-measures ANOVA on the 10MWT revealed a significant main effect of time (*F* = 84.052, *p* < 0.001). However, the main effect of group was not significant (*F* = 0.460, *p* = 0.053), and the group × time interaction was also not significant (*F* = 0.128, *p* = 0.993) (Table 6). No statistically significant differences were observed in the 10MWT comparison between groups at each evaluation time.

Analysis of within-group changes revealed significant differences across all groups in a repeated-measures ANOVA (Table 7). The *F*-value for EG 1 was 30.453 (*p* < 0.001), 21.980 (*p* < 0.001) for EG 2, and 34.369 (*p* < 0.001) for EG 3. All three groups showed a significant decrease in walking time over time in the 10MWT test. Post hoc tests showed that in all three groups, pre-test time was significantly higher than at weeks 2, 4, and 6, and at week 2, the average time was significantly higher than at weeks 4 and 6. Additionally, in all three groups, the 4-week time was significantly higher than the 6-week time, showing a gradual decrease in performance time as time progressed.

As a result of the analysis of 6MWT to determine walking endurance, the main effect of time was significant (*F* = 58.373, *p* < 0.001) (Table 8). On the other hand, the main effect of group was not significant (*F* = 1.513, *p* = 0.230), and the group × time interaction was also not significant (*F* = 1.405, *p* = 0.216). No statistically significant differences were observed in the 6MWT comparison between groups at each evaluation time.

Within-group changes were examined, and repeated-measures ANOVAs were significant for each group (Table 9). EG 1 had a *F* = 32.998 (*p* < 0.001), EG 2 had a *F* = 17.180 (*p* < 0.001), and EG 3 had a *F* = 19.715 (*p* < 0.001), demonstrating significant increases in 6MWT walking distance over time in all three groups. Post hoc analyses revealed similar patterns in all three groups. Specifically, 6MWT distance significantly increased at weeks 2, 4, and 6 compared to pre-test. Furthermore, there was a significant increase at weeks 4 and 6 compared to week 2, and a significant increase at week 6 compared to week 4. This indicates a gradual increase in walking distance as the measurement progressed.

Repeated-measures ANOVA on DGI showed a significant main effect of time (*F* = 114.385, *p* < 0.001) (Table 10). On the other hand, the main effect of group was not significant (*F* = 0.368, *p* = 0.694), and the group × time interaction was also not significant (*F* = 0.599, *p* = 0.731). No statistically significant differences were observed in the DGI comparison between groups at each evaluation time.

Repeated-measures ANOVAs showed significant within-group changes over time for all three groups (EG 1: *F* = 43.600, *p* < 0.001; EG 2: *F* = 34.040, *p* < 0.001; EG 3: *F* = 39.201, *p* < 0.001) (Table 11). Post-test results showed similar patterns across all three groups. Specifically, DGI scores significantly increased at weeks 2, 4, and 6 compared to pre-test. Furthermore, there was a significant increase at weeks 4 and 6 compared to week 2, and a significant increase at week 6 compared to week 4. This confirms a trend of gradual improvement in DGI as the measurement time progresses.

In this study, the repeated-measures analysis of variance for the BBS assessment to examine balance ability showed a significant main effect of time (*F* = 148.078, *p* < 0.001) (Table 12). On the other hand, the main effect of group was not significant (*F* = 0.979, *p* = 0.383), and the group × time interaction was also not significant (*F* = 0.931, *p* = 0.475). No statistically significant differences were observed in the BBS comparison between groups at each evaluation time.

The results of the repeated-measures ANOVA analysis examining within-group changes were statistically significant for all three groups (Table 13) (EG 1: *F* = 45.261, *p* < 0.001; EG 2: *F* = 63.915, *p* < 0.001; EG 3: *F* = 48.368, *p* < 0.001). Post hoc tests showed significant increases in scores at weeks 2, 4, and 6 compared to pre-test scores in all three groups. Significant increases were observed at weeks 4 and 6 compared to week 2, and at week 6 compared to week 4, confirming a gradual improvement in BBS over time.

Detailed objective dose metrics from robot logs were not consistently available and therefore were not reported. Detailed adherence metrics (planned vs. completed robot sessions and conventional therapy sessions) were not systematically recorded and therefore could not be reported. Participant tolerability was monitored throughout training (fatigue, soreness/pain, dizziness, and skin condition), and sessions could be paused or stopped if needed. However, adverse events and stopped sessions were not systematically recorded in a structured log, and therefore detailed quantitative counts by event category cannot be provided.

## 4. Discussion

### 4.1. Key Findings and Their Interpretation

Across all three groups, repeated-measures analyses demonstrated significant time effects for FAC, 10MWT, 6MWT, TUG, DGI, and BBS over the six-week intervention. However, neither the main effect of group nor the group-by-time interaction reached statistical significance for any outcome, indicating no detectable differential trajectories across robot types in this sample. For gait speed after stroke, an increase of approximately 0.16 m/s has been proposed as a minimal clinically important difference in the early/subacute period, particularly among individuals with low baseline gait speed. In the present study, mean 10MWT speed increased by approximately 0.16~0.17 m/s from baseline to week 6 across groups, suggesting that the observed time effects may reflect clinically meaningful improvement in walking performance, although MCID estimates may vary by population and baseline severity.

Stroke damages the central nervous system and can produce muscle weakness, spasticity, impaired coordination, and sensory and cognitive deficits, which collectively compromise gait and balance and restrict activities of daily living and social participation [27]. Because neuroplasticity and spontaneous recovery are relatively prominent in the acute and subacute stages, the intensity and frequency of rehabilitation during this period can influence the trajectory of functional recovery [28]. However, conventional therapy alone may not consistently deliver sufficient repetitive practice or standardized intensity progression, particularly for patients with severe deficits or high fall risk requiring enhanced safety management [7,8].

In particular, the improvements observed in this study are more likely to be interpreted as changes in functional activity levels rather than simply changes in impairment level (muscle strength, range of motion). The increase in FAC suggests the possibility of an increase in walking independence, and the decrease in the 10MWT and TUG (only EG1 and EG2 groups) performance times may be linked to improved mobility and transfer ability [29]. Furthermore, the increase in 6MWT suggests an increase in walking endurance and actual distance traveled, while the increases in DGI and BBS may be associated with improvements in balance and gait adaptation [30]. In other words, the time effect is significant because it goes beyond the statistical description of “improvement over time” and reflects clinical changes in the patient’s ability to perform daily functions. These function-focused results are highly consistent with the clinical hypothesis that robotic gait rehabilitation can contribute to the restoration of actual gait performance and balance control beyond simply providing repetition of gait patterns [31].

Non-significant between-group differences across the three robotic modalities should not be interpreted as device equivalence, because this study was not designed as an equivalence or non-inferiority trial. The absence of detectable differences may reflect limited power to identify realistic between-device effects in early post-stroke rehabilitation and the short follow-up time.

### 4.2. Clinical Implications

Against this backdrop, robot-assisted rehabilitation therapy is attracting attention as an intervention that can provide the task-oriented, repetitive training necessary for gait relearning in a relatively safe and consistent manner [15]. The robot system offers the advantage of standardizing treatment by providing highly reproducible movements according to preset trajectories, speeds, and levels of assistance. Furthermore, it quantitatively records the number of repetitions and the amount of exercise performed, allowing for systematic management of training volume [32]. Therefore, the overall functional improvements observed over time in this study, along with the characteristics of early post-stroke recovery, can be interpreted as supporting the clinical utility of the high-repetition, standardized training provided by robot-assisted training. The significant main effects of duration observed in all key gait and balance measures in this study are consistent with the typical recovery trajectory observed in acute stroke patients [33]. Since spontaneous recovery during this time is relatively rapid due to post-injury neuroplastic reorganization, intensive rehabilitation applied during this time may lead to improvements in functional activity over time [34]. These functional improvements over time are consistent with the clinically anticipated benefits of robotic-assisted gait training, such as functional recovery through repetition, intensity, and task-focused training [15].

Nevertheless, the lack of significant differences between types in this study suggests that in actual clinical applications, training dose (repetitions, intensity, assistance), the initial functional level of the subject, and the composition of the training task may have a greater impact on outcome variability than structural differences in the devices. In other words, even with different robots, functional improvements may be common if repeated gait training is provided over a certain period of time.

### 4.3. Comparison with Prior Studies

In particular, a meta-analysis that synthesized the effects of robotic gait training reported that robotic gait training can induce additional improvements in gait-related indicators when combined with conventional treatment. For example, a meta-analysis by Moucheboeuf et al. (2020) suggested that robotic gait training, when combined with standard rehabilitation and weight-bearing training, resulted in improvements in gait speed, FAC, and BBS [35]. This comprehensive evidence shares the same interpretive context as the present study, which showed consistent improvements in FAC, 6MWT, DGI, and BBS, and decreases in 10MWT and TUG (only EG1 and EG2 groups) times over time.

Meanwhile, the lack of a clear main effect between groups and a group × time interaction in this study partially aligns with previous studies suggesting that clinical outcomes may not necessarily differ significantly across robotic device types. Indeed, Sarı (2021) reported that the superiority between end-effector and exoskeleton devices was unclear [36]. Furthermore, a systematic review by Bruni et al. (2018) suggested that the effects of various robotic devices may be similar across key outcomes, supporting the possibility that training intensity, repetition volume, and subject characteristics (skill level, timing) may play a greater role in outcome variability than device type [37].

However, rather than concluding that device type is completely unimportant, a balanced discussion is needed, including evidence on which devices are likely to be more beneficial in which patient groups and at which time points. For example, a systematic review by Mehrholz and Pohl (2012) reported that the rate of achieving independent ambulation was higher with end-effector-based training [38]. A recent meta-analysis by Lee and Kim (2025) also found that end-effector devices were associated with relatively consistent improvements in gait speed in the subacute setting, providing room for interpreting device selection from the perspective of tailored application based on patient timing and functional level [39].

Furthermore, regarding the wearable exoskeleton included in this study, task orientation in an overhead environment is suggested as an advantage, but the evidence varies across outcome domains. For example, a meta-analysis by Hsu et al. (2023) reported that wearable exoskeleton gait training improved walking speed and balance ability compared to a control group [40]. Conversely, a meta-analysis by Lee et al. (2024) on an overhead robot exoskeleton demonstrated relatively strong evidence for improved walking speed, but inconsistent effects on balance and motor function [41]. This provides evidence for the “significant improvement over time, but limited differences between robot types” pattern in this study. That is, the expected effects of wearable devices may be more pronounced for certain outcomes influenced by factors such as speed and environmental adaptation, or conversely, the effects may be diluted by factors that may influence study design, such as modulation of exercise volume and distribution of subjects’ functional levels.

Furthermore, considering the mechanical differences between device types, the end-effector robot can repeatedly train the spatiotemporal characteristics of gait, such as stride length, gait cycle, and rhythm, as well as the weight-bearing pattern, by inducing the chain joint movements of the lower limbs through the motion trajectory of the footplate [18,36,42]. Fixed exoskeleton, on the other hand, offer advantages in early standing and normalizing gait patterns in acute or subacute critically ill patients due to their ease of weight support and alignment [11,19]. Wearable exoskeletons offer the advantage of enabling training involving realistic tasks such as turning and obstacle avoidance in an overground environment [40].

### 4.4. Study Limitations and Future Research Directions

Although session time was standardized, time-matching does not ensure comparable delivered dose across devices, and incomplete device-exported logs limited objective quantification of dose.

This study has several limitations. First, it was conducted at a single institution with a modest sample size. Second, all groups received intensive conventional PT/OT in parallel with robot training, limiting causal isolation of robot-type effects and allowing spontaneous recovery and standard rehabilitation to contribute to improvements. Third, although session duration was standardized (40 min), time-matching does not guarantee dose equivalence across devices or participants because therapist-individualized body-weight support, speed, assistance level, and step targets for safety and capability, and complete device-exported dose logs were not uniformly available. In addition, adverse events and session interruptions were monitored clinically but were not systematically captured in a structured dataset, limiting quantitative safety reporting. Additionally, the study was not prospectively trial-registered and was not planned under CONSORT reporting, which may limit transparency compared with fully registered randomized trials. Finally, the follow-up period was relatively short and some neurological descriptors were not consistently available, which may limit generalizability. Because repeated-measures ANOVA was implemented as a complete-case analysis, results may be sensitive to attrition; linear mixed-effects modeling would be more robust for incomplete follow-up. Future multicenter trials with larger samples and stratified randomization by baseline functional level should quantify intervention dose using standardized metrics (step count, distance, speed, body-weight support, assistance level, and active participation) and include longer-term outcomes such as community walking, falls, participation, and quality of life.

## 5. Conclusions

This study compared the clinical effectiveness of robot-assisted gait rehabilitation using end-effectors (EG 1), fixed exoskeletons (EG 2), and wearable exoskeletons (EG 3) in early post-stroke patients. The results showed significant improvements over time in key gait and balance measures, including FAC, 10MWT, 6MWT, TUG, DGI, and BBS, suggesting that robot-assisted gait rehabilitation can be a useful intervention for functional recovery in early post-stroke patients. Conversely, the group main effect and group × time interaction were insignificant in all assessments, indicating that statistically significant differences in functional improvement across robot types were not evident under the conditions of this study. Because this was not an equivalence/non-inferiority trial, these non-significant findings should not be interpreted as evidence of device equivalence, but rather as not detectable between-device differences in this sample and clinical context. Given the possibility of limited power for between-group comparisons, these findings should be interpreted cautiously, and larger studies are warranted to determine whether clinically meaningful between-device differences exist. Therefore, clinical practice suggests that device selection should be tailored to the patient’s initial functional level, safety, treatment goals, and operating conditions, rather than focusing on the superiority of a particular robot.

## Figures and Tables

**Table 1:** General characteristics and homogeneity test of the subjects.

	EG 1 (*n* = 19)	EG 2 (*n* = 17)	EG 3 (*n* = 18)	*F*/χ^2^	*p*
Age (years)	50.88 ± 11.78	55.70 ± 17.07	53.73 ± 14.41	0.488	0.617
Height (cm)	170.44 ± 6.94	170.82 ± 8.21	169.78 ± 6.64	0.103	0.902
Weight (kg)	64.80 ± 6.19	63.73 ± 7.91	63.23 ± 6.66	0.245	0.784
MMSE (score)	24.83 ± 2.52	24.76 ± 3.43	24.84 ± 2.98	0.004	0.996
Onset time (days)	48.16 ± 21.47	40.82 ± 21.42	39.72 ± 23.03	0.802	0.457
Stroke type (Ischemic/Hemorrhagic)	12/7	12/5	12/6	0.226	0.895
Lesion side (Left/Right)	14/5	12/5	12/6	0.224	0.896
NIHSS (score)	10.16 ± 4.48	11.00 ± 5.48	10.67 ± 5.20	0.138	0.877
FMA-LE (score)	19.53 ± 3.86	17.82 ± 3.36	20.61 ± 6.40	1.734	0.193
MAS ^†^ (grade)	1 [1–1.25]	1 [1–1.50]	1 [1–1.00]	0.257	0.882
FAC (score)	1.16 ± 0.38	1.23 ± 0.43	1.21 ± 0.41	0.124	0.883
10MWT (m/s)	0.30 ± 0.16	0.35 ± 0.23	0.26 ± 0.15	1.539	0.267
6MWT (meter)	89.72 ± 26.42	111.47 ± 72.66	101.05 ± 52.27	0.726	0.489
TUG (score)	40.83 ± 11.57	40.55 ± 14.11	39.93 ± 16.42	0.012	0.988
DGI (score)	6.22 ± 2.86	7.82 ± 4.31	6.89 ± 3.58	0.859	0.430
BBS (score)	24.27 ± 8.07	28.29 ± 11.94	25.84 ± 9.66	0.718	0.492

Abbreviations: EG 1: end-effector group; EG 2: fixed exoskeleton group; EG 3: wearable exoskeleton group; NIHSS: national institutes of health stroke scale; FMA-LE: Fugl–Meyer assessment-lower extremity; MAS: modified Ashworth scale; MMSE: mini-mental state examination; FAC: functional ambulation category; 10MWT: 10-m walk test; 6MWT: six-minute walk test; TUG: timed up-and-go test; DGI: dynamic gait index; BBS: Berg Balance Scale. Notes: data are reported as mean ± SD, ^†^: MAS presented as median using numeric coding (1+ coded as 1.5).

**Table 2:** Statistical analysis of TUG using repeated-measures analysis of variance.

S	SS	df	MS	*F*	*p*	ηp^2^
Time	3427.688	3	1142.563	18.193	<0.001 **	0.423
Group	43.328	2	20.664	0.023	0.977	0.000
Group × Time	83.532	6	13.922	0.222	0.969	0.102

** *p* < 0.001.

**Table 3:** Comparative analysis of TUG evaluations within groups.

	EG 1 (*n* = 19)	EG 2 (*n* = 17)	EG 3 (*n* = 18)
Pre-test	40.83 ± 11.57	40.55 ± 14.11	39.93 ± 16.42
2 weeks	36.87 ± 11.22	34.29 ± 16.25	35.15 ± 17.31
4 weeks	33.73 ± 11.22	33.12 ± 16.64	31.99 ± 14.36
6 weeks	29.21 ± 8.56	28.73 ± 5.91	30.42 ± 19.48
mean (95% CI)	11.51 [8.70–14.63]	5.90 [0.87–10.92]	9.50 [5.69–13.32]
*F*	36.050	2.514	14.128
*p*	<0.001 **	0.069	<0.001 **
Post hoc	Pre > 2, 4, 6	Pre > 2, 4, 6	Pre > 2, 4, 6
	2 > 4, 6	2, 4 > 6	2 > 4, 6
	4 > 6		

Abbreviations: EG 1: end-effector group; EG 2: fixed exoskeleton group; EG 3: wearable exoskeleton group; TUG: timed up-and-go test; ** *p* < 0.001. Notes: data are reported as mean ± SD.

**Table 4:** Statistical analysis of FAC using repeated-measures analysis of variance.

S	SS	df	MS	*F*	*p*	ηp^2^
Time	42.415	3	14.138	80.528	<0.001 **	0.612
Group	0.670	2	0.335	0.382	0.684	0.015
Group × Time	0.249	6	0.041	0.236	0.921	0.009

** *p* < 0.001.

**Table 5:** Comparative analysis of FAC evaluations within groups.

	EG 1 (*n* = 19)	EG 2 (*n* = 17)	EG 3 (*n* = 18)
Pre-test	1.16 ± 0.38	1.23 ± 0.43	1.21 ± 0.41
2 weeks	1.77 ± 0.42	1.82 ± 0.63	1.84 ± 0.50
4 weeks	2.00 ± 0.48	2.17 ± 0.80	2.10 ± 0.65
6 weeks	2.27 ± 0.46	2.52 ± 0.94	2.42 ± 0.69
*F*	27.530	24.920	28.680
*p*	<0.001 **	<0.001 **	<0.001 **
Post hoc	Pre < 2, 4, 6	Pre < 2, 4, 6	Pre < 2, 4, 6
	2 < 4, 6	2 < 4, 6	2 < 4, 6
	4 < 6	4 < 6	4 < 6

Abbreviations: EG 1: end-effector group; EG 2: fixed exoskeleton group; EG 3: wearable exoskeleton group; FAC: functional ambulation category; ** *p* < 0.001. Notes: data are reported as mean ± SD.

**Table 6:** Statistical analysis of 10MWT using repeated-measures analysis of variance.

S	SS	df	MS	*F*	*p*	ηp^2^
Time	0.825	3	0.275	84.052	<0.001 **	0.032
Group	0.919	2	0.460	3.124	0.053	0.003
Group × Time	0.003	6	0.001	0.128	0.993	0.047

** *p* < 0.001.

**Table 7:** Comparative analysis of 10MWT evaluations within groups.

	EG 1 (*n* = 19)	EG 2 (*n* = 17)	EG 3 (*n* = 18)
Pre-test	0.30 ± 0.16	0.35 ± 0.23	0.26 ± 0.15
2 weeks	0.36 ± 0.18	0.46 ± 0.24	0.29 ± 0.21
4 weeks	0.42 ± 0.21	0.52 ± 0.27	0.36 ± 0.13
6 weeks	0.46 ± 0.22	0.55 ± 0.31	0.39 ± 0.10
*F*	30.453	21.980	34.369
*p*	<0.001 **	<0.001 **	<0.001 **
Post hoc	Pre > 2, 4, 6	Pre > 2, 4, 6	Pre > 2, 4, 6
	2 > 4, 6	2 > 4, 6	2 > 4, 6
	4 > 6	4 > 6	4 > 6

Abbreviations: EG 1: end-effector group; EG 2: fixed exoskeleton group; EG 3: wearable exoskeleton group; 10MWT: 10-m walk test; ** *p* < 0.001. Notes: data are reported as mean ± SD.

**Table 8:** Statistical analysis of 6MWT using repeated-measures analysis of variance.

S	SS	df	MS	*F*	*p*	ηp^2^
Time	149,778.344	3	49,926.115	58.373	<0.001 **	0.531
Group	52,568.468	2	26,284.234	1.513	0.230	0.056
Group × Time	7208.131	6	1201.355	1.405	0.216	0.052

** *p* < 0.001.

**Table 9:** Comparative analysis of 6MWT evaluations within groups.

	EG 1 (*n* = 19)	EG 2 (*n* = 17)	EG 3 (*n* = 18)
Pre-test	89.72 ± 26.42	111.47 ± 72.66	101.05 ± 52.27
2 weeks	121.83 ± 35.18	149.88 ± 76.29	134.00 ± 56.78
4 weeks	131.66 ± 46.52	181.70 ± 98.39	150.15 ± 74.13
6 weeks	146.44 ± 54.31	201.05 ± 96.25	167.68 ± 86.81
*F*	32.998	17.180	19.715
*p*	<0.001 **	<0.001 **	<0.001 **
Post hoc	Pre < 2, 4, 6	Pre < 2, 4, 6	Pre < 2, 4, 6
	2 < 4, 6	2 < 4, 6	2 < 4, 6
	4 < 6	4 < 6	4 < 6

Abbreviations: EG 1: end-effector group; EG 2: fixed exoskeleton group; EG 3: wearable exoskeleton group; 6MWT: six-minute walk test; ** *p* < 0.001. Notes: data are reported as mean ± SD.

**Table 10:** Statistical analysis of DGI using repeated-measures analysis of variance.

S	SS	df	MS	*F*	*p*	ηp^2^
Time	1068.478	3	356.159	114.385	<0.001 **	0.692
Group	76.846	2	38.423	0.368	0.694	0.014
Group × Time	11.194	6	1.866	0.599	0.731	0.023

** *p* < 0.001.

**Table 11:** Comparative analysis of DGI evaluations within groups.

	EG 1 (*n* = 19)	EG 2 (*n* = 17)	EG 3 (*n* = 18)
Pre-test	6.22 ± 2.86	7.82 ± 4.31	6.89 ± 3.58
2 weeks	10.22 ± 5.27	10.94 ± 5.47	10.52 ± 5.31
4 weeks	11.16 ± 5.77	12.47 ± 5.75	11.68 ± 5.69
6 weeks	11.83 ± 6.15	14.11 ± 6.33	12.78 ± 6.23
*F*	43.600	34.040	39.201
*p*	<0.001 **	<0.001 **	<0.001 **
Post hoc	Pre < 2, 4, 6	Pre < 2, 4, 6	Pre < 2, 4, 6
	2 < 4, 6	2 < 4, 6	2 < 4, 6
	4 < 6	4 < 6	4 < 6

Abbreviations: EG 1: end-effector group; EG 2: fixed exoskeleton group; EG 3: wearable exoskeleton group; DGI: dynamic gait index; ** *p* < 0.001. Notes: data are reported as mean ± SD.

**Table 12:** Statistical analysis of BBS using repeated-measures analysis of variance.

S	SS	df	MS	*F*	*p*	ηp^2^
Time	4894.331	3	1631.444	148.078	<0.001 **	0.745
Group	569.498	2	284.749	0.979	0.383	0.037
Group × Time	61.522	6	10.254	0.931	0.475	0.035

** *p* < 0.001.

**Table 13:** Comparative analysis of BBS evaluations within groups.

	EG 1 (*n* = 19)	EG 2 (*n* = 17)	EG 3 (*n* = 18)
Pre-test	24.27 ± 8.07	28.29 ± 11.94	25.84 ± 9.66
2 weeks	31.00 ± 7.21	36.35 ± 8.32	33.05 ± 7.84
4 weeks	34.33 ± 8.90	39.11 ± 8.84	36.21 ± 9.12
6 weeks	37.88 ± 9.11	39.76 ± 9.19	38.57 ± 8.99
*F*	45.261	63.915	48.368
*p*	<0.001 **	<0.001 **	<0.001 **
Post hoc	Pre < 2, 4, 6	Pre < 2, 4, 6	Pre < 2, 4, 6
	2 < 4, 6	2 < 4, 6	2 < 4, 6
	4 < 6	4 < 6	4 < 6

Abbreviations: EG 1: end-effector group; EG 2: fixed exoskeleton group; EG 3: wearable exoskeleton group; BBS: Berg Balance Scale; ** *p* < 0.001. Notes: data are reported as mean ± SD.

## Data Availability

The original contributions presented in this study are included in the article. Further inquiries can be directed to the corresponding author.

## References

[1] Feigin V.L., Brainin M., Norrving B., Martins S.O., Pandian J., Lindsay P., F Grupper M., Rautalin I. (2025). World Stroke Organization: Global Stroke Fact Sheet 2025. Int. J. Stroke.

[2] Mariana de Aquino Miranda J., Mendes Borges V., Bazan R., José Luvizutto G., Sabrysna Morais Shinosaki J. (2023). Early Mobilization in Acute Stroke Phase: A Systematic Review. Top. Stroke Rehabil..

[3] O’Dell M.W. (2023). Stroke Rehabilitation and Motor Recovery. Continuum.

[4] Lee K.E., Choi M., Jeoung B. (2022). Effectiveness of Rehabilitation Exercise in Improving Physical Function of Stroke Patients: A Systematic Review. Int. J. Env. Res. Public Health.

[5] Rigual R., Fuentes B., Díez-Tejedor E. (2023). Management of Acute Ischemic Stroke. Med. Clin..

[6] García-Bouyssou I., Laredo C., Massons M., Serrano M., Moreira F., Cabero-Arnold A., Urra X., Chamorro A. (2024). Clinical and Neuroanatomical Predictors of Post-Stroke Fatigue. J. Stroke Cerebrovasc. Dis..

[7] Almutairi S.M., Alfouzan M.M., Almutairi T.S., Alkaabi H.A., AlMulaifi M.T., Almutairi M.K., Alhuthaifi F.K., Swank C. (2023). The Safety and Feasibility of Lower Body Positive Pressure Treadmill Training in Individuals with Chronic Stroke: An Exploratory Study. Brain Sci..

[8] Hung S.H., Ackerley S., Connell L.A., Bayley M.T., Best K.L., Donkers S.J., Dukelow S.P., Ezeugwu V.E., Milot M.-H., Peters S. (2025). Real-World Experiences of Therapy Staff Implementing an Intensive Rehabilitation Protocol in Canadian Stroke Inpatient Rehabilitation Settings: A Multi-Site Survey Study. Phys. Ther..

[9] Yeung L.-F., Lau C.C.Y., Lai C.W.K., Soo Y.O.Y., Chan M.-L., Tong R.K.Y. (2021). Effects of Wearable Ankle Robotics for Stair and Over-Ground Training on Sub-Acute Stroke: A Randomized Controlled Trial. J. Neuroeng. Rehabil..

[10] Marek K., Górski J., Karolczyk P., Redlicka J., Zubrycki I., Miller E. (2025). Feasibility of Wearable Devices for Motivating Post-Stroke Patients. Sensors.

[11] Calafiore D., Negrini F., Tottoli N., Ferraro F., Ozyemisci-Taskiran O., de Sire A. (2022). Efficacy of Robotic Exoskeleton for Gait Rehabilitation in Patients with Subacute Stroke: A Systematic Review. Eur. J. Phys. Rehabil. Med..

[12] Takebayashi T., Takahashi K., Amano S., Gosho M., Sakai M., Hashimoto K., Hachisuka K., Uchiyama Y., Domen K. (2022). Robot-Assisted Training as Self-Training for Upper-Limb Hemiplegia in Chronic Stroke: A Randomized Controlled Trial. Stroke.

[13] Zhang H., Li X., Gong Y., Wu J., Chen J., Chen W., Pei Z., Zhang W., Dai L., Shu X. (2023). Three-Dimensional Gait Analysis and sEMG Measures for Robotic-Assisted Gait Training in Subacute Stroke: A Randomized Controlled Trial. Biomed. Res. Int..

[14] Winterbottom L., Nilsen D.M. (2024). Motor Learning Following Stroke: Mechanisms of Learning and Techniques to Augment Neuroplasticity. Phys. Med. Rehabil. Clin. N. Am..

[15] Koldaş Doğan Ş. (2024). Robot-Assisted Gait Training in Stroke. Turk. J. Phys. Med. Rehabil..

[16] Kolobe T.H.A., Fagg A.H. (2019). Robot Reinforcement and Error-Based Movement Learning in Infants With and Without Cerebral Palsy. Phys. Ther..

[17] Banyai A.D., Brișan C. (2024). Robotics in Physical Rehabilitation: Systematic Review. Healthcare.

[18] Lee J., Kim D.Y., Lee S.H., Kim J.H., Kim D.Y., Lim K.-B., Yoo J. (2023). End-Effector Lower Limb Robot-Assisted Gait Training Effects in Subacute Stroke Patients: A Randomized Controlled Pilot Trial. Medicine.

[19] Zhu Y.-H., Ruan M., Yun R.-S., Zhong Y.-X., Zhang Y.-X., Wang Y.-J., Sun Y.-L., Cui J.-W. (2023). Is Leg-Driven Treadmill-Based Exoskeleton Robot Training Beneficial to Poststroke Patients: A Systematic Review and Meta-Analysis. Am. J. Phys. Med. Rehabil..

[20] Marinaro C., Muglia L., Squartecchia S., Cozza A., Corsonello A., Pranno L., Ferrarin M., Lencioni T. (2025). Mapping the Role of Robot-Assisted Gait Training in Post-Stroke Recovery Among Elderly Patients: A Scoping Review. J. Clin. Med..

[21] Cinnera A.M., Marrano S., De Bartolo D., Iosa M., Bisirri A., Leone E., Stefani A., Koch G., Ciancarelli I., Paolucci S. (2023). Convergent Validity of the Timed Walking Tests with Functional Ambulatory Category in Subacute Stroke. Brain Sci..

[22] Tarihci Cakmak E., Yaliman A., Torna G., Sen E.I. (2024). The Effectiveness of Bodyweight-Supported Treadmill Training in Stroke Patients: Randomized Controlled Trial. Neurol. Sci..

[23] Lattouf N.A., Tomb R., Assi A., Maynard L., Mesure S. (2021). Eccentric Training Effects for Patients with Post-Stroke Hemiparesis on Strength and Speed Gait: A Randomized Controlled Trial. NeuroRehabilitation.

[24] Choi Y., Bae Y., Cha B., Ryu J. (2022). Deep Learning-Based Subtask Segmentation of Timed Up-and-Go Test Using RGB-D Cameras. Sensors.

[25] Batool S., Zafar H., Gilani S.A., Ahmad A., Hanif A. (2023). Intrarater and Interrater Reliability of the Dynamic Gait Index in Post Stroke Patients with Eye Movement Disorders. J. Bodyw. Mov. Ther..

[26] Zhang Y., Zhao W., Wan C., Wu X., Huang J., Wang X., Huang G., Ding W., Chen Y., Yang J. (2024). Exoskeleton Rehabilitation Robot Training for Balance and Lower Limb Function in Sub-Acute Stroke Patients: A Pilot, Randomized Controlled Trial. J. Neuroeng. Rehabil..

[27] Potter T.B.H., Tannous J., Vahidy F.S. (2022). A Contemporary Review of Epidemiology, Risk Factors, Etiology, and Outcomes of Premature Stroke. Curr. Atheroscler. Rep..

[28] Mah S.M., Goodwill A.M., Seow H.C., Teo W.-P. (2022). Evidence of High-Intensity Exercise on Lower Limb Functional Outcomes and Safety in Acute and Subacute Stroke Population: A Systematic Review. Int. J. Environ. Res. Public Health.

[29] Sczesny-Kaiser M., Trost R., Aach M., Schildhauer T.A., Schwenkreis P., Tegenthoff M. (2019). A Randomized and Controlled Crossover Study Investigating the Improvement of Walking and Posture Functions in Chronic Stroke Patients Using HAL Exoskeleton—The HALESTRO Study (HAL-Exoskeleton STROke Study). Front. Neurosci..

[30] Neves M.V.M., Furlan L., Fregni F., Battistella L.R., Simis M. (2023). Robotic-Assisted Gait Training (RAGT) in Stroke Rehabilitation: A Pilot Study. Arch. Rehabil. Res. Clin. Transl..

[31] Do J., Lim W.-T., Kim D.Y., Ko E.J., Ko M.-H., Kim G.W., Kim J.H., Kim S., Kim H. (2024). Effects of High-Intensity Interval Robot-Assisted Gait Training on Cardiopulmonary Function and Walking Ability in Chronic Stroke Survivors: A Multicenter Single-Blind Randomized Controlled Trial. J. Back. Musculoskelet. Rehabil..

[32] Hu M.-M., Wang S., Wu C.-Q., Li K.-P., Geng Z.-H., Xu G.-H., Dong L. (2024). Efficacy of Robot-Assisted Gait Training on Lower Extremity Function in Subacute Stroke Patients: A Systematic Review and Meta-Analysis. J. Neuroeng. Rehabil..

[33] Rajashekar D., Boyer A., Larkin-Kaiser K.A., Dukelow S.P. (2024). Technological Advances in Stroke Rehabilitation: Robotics and Virtual Reality. Phys. Med. Rehabil. Clin. N. Am..

[34] Dromerick A.W., Geed S., Barth J., Brady K., Giannetti M.L., Mitchell A., Edwardson M.A., Tan M.T., Zhou Y., Newport E.L. (2021). Critical Period After Stroke Study (CPASS): A Phase II Clinical Trial Testing an Optimal Time for Motor Recovery after Stroke in Humans. Proc. Natl. Acad. Sci. USA.

[35] Moucheboeuf G., Griffier R., Gasq D., Glize B., Bouyer L., Dehail P., Cassoudesalle H. (2020). Effects of Robotic Gait Training after Stroke: A Meta-Analysis. Ann. Phys. Rehabil. Med..

[36] Sarı A. (2021). Comparison of End-Effector and Exoskeleton Devices with Robot-Assisted Gait Training in Patients with Stroke. J. Surg. Med..

[37] Bruni M.F., Melegari C., De Cola M.C., Bramanti A., Bramanti P., Calabrò R.S. (2018). What Does Best Evidence Tell Us about Robotic Gait Rehabilitation in Stroke Patients: A Systematic Review and Meta-Analysis. J. Clin. Neurosci..

[38] Mehrholz J., Pohl M. (2012). Electromechanical-Assisted Gait Training after Stroke: A Systematic Review Comparing End-Effector and Exoskeleton Devices. J. Rehabil. Med..

[39] Lee J.H., Kim G. (2025). Effectiveness of Robot-Assisted Gait Training in Stroke Rehabilitation: A Systematic Review and Meta-Analysis. J. Clin. Med..

[40] Hsu T.-H., Tsai C.-L., Chi J.-Y., Hsu C.-Y., Lin Y.-N. (2023). Effect of Wearable Exoskeleton on Post-Stroke Gait: A Systematic Review and Meta-Analysis. Ann. Phys. Rehabil. Med..

[41] Lee M.-H., Tian M.-Y., Kim M.-K. (2024). The Effectiveness of Overground Robot Exoskeleton Gait Training on Gait Outcomes, Balance, and Motor Function in Patients with Stroke: A Systematic Review and Meta-Analysis of Randomized Controlled Trials. Brain Sci..

[42] Tanaka N., Yano H., Ebata Y., Ebihara K. (2023). Influence of Robot-Assisted Gait Training on Lower-Limb Muscle Activity in Patients with Stroke: Comparison with Conventional Gait Training. Ann. Rehabil. Med..

